# Contactless Electrocatheter Tracing within Human Body via Magnetic Sensing: A Feasibility Study

**DOI:** 10.3390/s22103880

**Published:** 2022-05-20

**Authors:** Emilio Andreozzi, Daniele Esposito, Paolo Bifulco

**Affiliations:** Department of Electrical Engineering and Information Technologies, University of Naples Federico II, Via Claudio, 21, 80125 Napoli, Italy; emilio.andreozzi@unina.it (E.A.); daniele.esposito@unina.it (D.E.)

**Keywords:** magnetic sensing, contactless sensing, electrocatheter, pacemaker implant, lead positioning

## Abstract

During surgical procedures, real-time estimation of the current position of a metal lead within the patient’s body is obtained by radiographic imaging. The inherent opacity of metal objects allows their visualization using X-ray fluoroscopic devices. Although fluoroscopy uses reduced radiation intensities, the overall X-ray dose delivered during prolonged exposure times poses risks to the safety of patients and physicians. This study proposes a potential alternative to real-time visualization of a lead inside the human body. In principle, by making a weak current flow through the lead and measuring the related magnetic field generated outside the body, it is possible to trace the position of the lead. This hypothesis was verified experimentally via two tests: one carried out on a curved copper wire in air and one carried out on a real pacemaker lead in a saline solution. In the second test, a pacemaker lead and a large return electrode were placed in a tank filled with a saline solution that reproduced the mean resistivity of the human torso. In both tests, a current flowed through the lead, which consisted of square pulses with short duration, to avoid any neuro-muscular stimulation effects in a real scenario. A small coil with a ferrite core was moved along a grid of points over a plastic sheet and placed just above the lead to sample the spatial amplitude distribution of the magnetic induction field produced by the lead. For each measurement point, the main coil axis was oriented along the *x* and *y* axes of the plane to estimate the related components of the magnetic induction field. The two matrices of measurements along the *x* and *y* axes were further processed to obtain an estimate of lead positioning. The preliminary results of this study support the scientific hypothesis since the positions of the leads were accurately estimated. This encourages to deepen the investigation and overcome some limitations of this feasibility study.

## 1. Introduction

During some surgical procedures such as pacemaker or defibrillator implantation or electrophysiology studies, the physician must visually perceive the position of a lead (a specific electrocatheter) inside the patient’s body in real-time. Typically, leads are inserted into the body using blood vessels as channels to reach specific locations within the patient, such as the heart cavities [1,2].

Currently, X-ray equipment is used that provides a low dose of radiation to follow the path of the lead inside the body. The lead made of metallic material is particularly radio-opaque compared to other body tissues and is easily recognizable in X-ray projections. However, for a correct introduction of the catheter, it is important to follow its progress in real-time, and it is necessary to keep the X-ray tube on throughout its insertion and positioning. Although the radiation used today is quite low, it is still harmful to the health of the patient and medical operators [3,4,5,6].

In fluoroscopic equipment, the radiation dose is reduced by using fewer X photons to form a single image. However, the reduction in the number of photons is intrinsically linked to an increase in quantum noise; this noise is the predominant noise in fluoroscopic images and is the main limitation to dose reduction [7]. Furthermore, the fluoroscopic equipment must necessarily remain in operation throughout the catheter placement procedure providing real-time feedback to the medical operator. This, of course, conflicts with the need to reduce the radiation dose absorbed both by the patient and even more so by the medical operator [8,9]. To try to reduce quantum noise while maintaining a low radiation dose, various image processing techniques have been proposed, and some of these are implemented by modern fluoroscopic systems. In particular, some studies present filtering in space and time that guarantee a conspicuous reduction of quantum noise and also the preservation of contrasting image details such as, for example, those related to a lead [10,11,12].

Therefore, it is of great interest to evaluate alternative techniques that can provide the position of a lead inside the patient’s body in real-time without the use of ionizing radiation. Many devices used for electrophysiology studies and ablation anti-tachyarrhythmias therapy use special catheters whose tips can be accurately localized when inside the cardiac cavities. As an example, the CARTO catheter mapping system (Biosense, Diamond Bar, CA, USA), one of the most widely used systems to map the endocardial potentials, utilizes a low-level magnetic field generated from three separate coils in a locator pad placed beneath the patient [13]. A specific sensor embedded in the tip of a specialized mapping catheter (e.g., NaviSTAR) measures the magnetic field strength from each coil, which is inversely proportional to the sensor-coil distance. Then, the catheter tip sensor is precisely located using triangulation in space [14]. Recently, the use of these devices has been preliminarily proposed to drastically reduce radiation dose during pacemaker and defibrillator lead implantation procedure procedures [15,16,17,18,19]. However, these techniques rely on the use of such special catheters, which are specific for electrophysiology and ablation studies.

Recently, implantable sensors are emerging with capabilities to monitor physiological parameters (e.g., temperature, blood pressure, oxygen level) to communicate wirelessly with an external device, and to be biodegradable, i.e., made of biocompatible materials that undergo controlled degradation over time, leaving nontoxic by-products that can be removed by metabolic activity [20,21,22,23]. Such transient implants do not have to be extracted from the body, thus minimizing the risk of infection. On the other hand, to date, wireless biodegradable implants are in their early state, and major challenges must be addressed. The fabrication of a fully bioresorbable implant (including power source, circuitry, and wires) is very difficult and the biocompatibility of the materials used (e.g., metals, polymers, and composites) and their potential toxicity must be deeply investigated (in terms of cytotoxicity and inflammatory response [21]) for possible future implementation in clinical practice; furthermore, the operational lifespan (hours-days) of currently available devices does not meet many clinical needs [20,21].

In a recent study, a contactless system for pacemaker pulse detection has been proposed [24]. The system is based on a single coil that senses the magnetic induction field generated by the pacemaker current pulses flowing through the electrocatheter, thus enabling contactless monitoring of pacemaker activity.

Such an operating principle stands as a further alternative solution for the estimation of electrocatheter positioning within the human body. Indeed, the amplitude of the electromotive force (EMF) induced on a coil by the magnetic induction field generated by a current wire varies with the coil orientation and distance from the wire. Hence, an array of coils could be used in principle to simultaneously sense the magnetic induction field in multiple sites over the human chest to determine the positioning of an electrocatheter excited by current pulses. Of course, the amplitude and duration of such current pulses must be appropriately set to avoid any undesired neuro-muscular stimulation.

This study aims to preliminarily test the capability of estimating the positioning of a common electrocatheter immersed in a saline solution via a single coil placed outside on a plane above the electrocatheter. The novelty of the proposed solution is that it does not require a sensorized catheter, thus applying to any common electrocatheter usually adopted for pacemaker/defibrillator implant and electrophysiological studies.

## 2. Materials and Methods

### 2.1. Principles of Operation

In previous studies [24] it has been shown that the pulse of the pacemaker conducted through the lead can be detected by a coil outside the body. The coil can passively monitor the magnetic field produced by the current pulse conducted by the pacemaker lead, the coil does not require to be in contact with the patient and still work when placed a few centimeters away from the body. Extrapolating the basic concept of the sensor operation to detect pacemaker pulses and making use of multiple coils placed in close proximity to the patient, it is, in principle, possible to detect the position of the lead inside the patient’s body. This study aims to practically verify the hypothesis of recognizing the placement of an entire catheter through a matrix of coils placed on a plane in the front of the patient.

Obviously, it is necessary to pass a current inside the lead to generate a magnetic field. However, one must be sure not to obtain any effect of electrostimulation or tissue heating. For this purpose, it was thought to use rectangular current pulses of extremely short duration, much shorter than the typical chronaxie times of nervous and muscular tissues. In this way, using even relatively high amplitudes of the pulse is not likely to produce any effect of electrostimulation as these pulses fall far below the strength-duration curve of the human tissues. The very short duration of the pulses will not give rise to any significant increase in temperature at the electrode tip or in any surrounding tissue.

Since the signal revealed by the coil is proportional to the first temporal derivative of the magnetic field produced by the current, the rising and falling edges of the pulse should be steep enough to generate a sufficient signal at the coil terminals.

**Figure 1:**
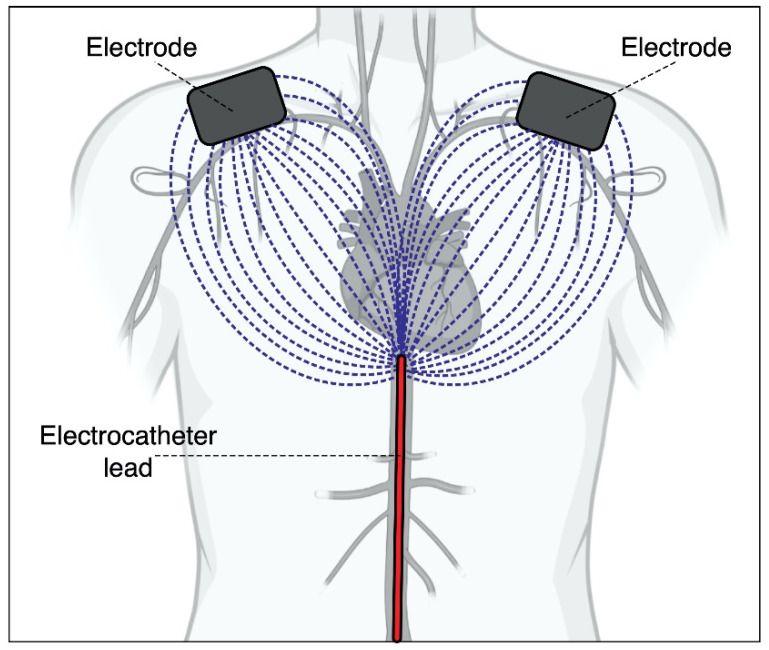
Graphic description of the operating principle. The current confined in the electrocatheter within the body produces a reasonable magnetic induction field that can be sensed from the outside via a coil. The amplitude of the coil signal would lower when moving away from the catheter and when the angle between the local catheter direction and the main coil axis decreases. The current flowing from the electrocatheter tip toward the return electrodes spreads through the tissues of the human torso into many different paths, which provide different, weak contributions to the magnetic induction field concatenated with the coil, i.e., a much lower coil signal. The spatial variations of the coil signal amplitude could therefore support the catheter localization within the body.

As the current passes through the electrode, it is no longer confined within the metallic wire but spreads throughout the body tissues and is collected by a large return electrode placed on the patient’s skin. The larger the return electrode, the lower the current density reaching it, and so the current density within the tissues located between the two electrodes. The distribution of the local ionic current inside the tissues will obviously depend on the electrical properties of each tissue, but overall, it will tend to distribute itself over a wide area largely by reducing its density. The current will be distributed radially within the tissues close to the electrode tip, as describing a wide cone-beam covering a very large solid angle. The total current through the body consists of many current threads dispersed inside the body, each of these will give a contribution to the external magnetic field, making it lower in intensity and uniform outside the body (see Figure 1).

To generate at least one image of the catheter-like that offered by fluoroscopy, i.e., an X-ray projection, it is necessary to measure the contribution of the magnetic field in a plane located frontally to the patient’s body in the two directing axes of the plane. The magnetic field component perpendicular to this plane could provide information about the three-dimensional arrangement of the wire within the patient, but in this initial study, it was not taken into account by limiting the analysis to the projection of the catheter in the frontal plane.

### 2.2. Experimental Setup

Only one coil was actually used for the preliminary tests instead of an array of coils or a matrix. The coil had a cylindrical ferrite core, with a diameter of 4 mm and a length of 29 mm, of which only 17 mm were actually covered by the coil turns. The coil had an inductance of 5.60 mH, measured at 2 kHz via an LCR meter (LCR-816, Good Will Instrument Co., Ltd., No. 7-1, Jhongsing Road., Tucheng Dist., New Taipei City, Taiwan) and a resistance of 12.2 Ω.

The single-coil was moved to various measuring positions arranged in a grid over the surface of a measurement plane. For each measurement point, the voltage induced on the coil was recorded twice, i.e., by orienting the main axis of the coil along the *x* and *y* axes of the measurement plane.

**Figure 2:**
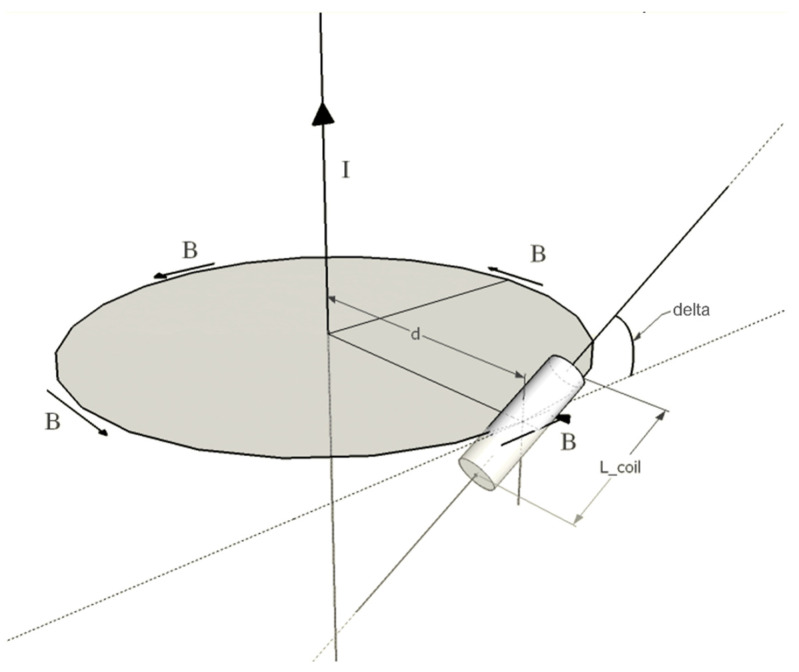
Magnetic induction field generated by an infinite current wire, which concatenates with a coil. The wire represents the lead inside the patient, while the dotted line lays on the measurement plane.

As reported in [24], the amplitude of the electromotive force (EMF) induced in a coil by an infinitely long wire with a square pulse current (see Figure 2) can be expressed as:

(1) $$\begin{array}{c} \left|\mathrm{EMF}\right|=S\frac{\mu_{0}\mu_{r}}{2\pi d}\frac{I_{STIM}}{t_{RISE}}\overset{N}{\underset{k=1}{\sum }}\cos\delta_{k} \end{array}$$

where *N* is the number of coil loops, *S* is the surface area of the coil loops, $\mu_{0}$ and $\mu_{r}$ are, respectively, the vacuum magnetic permeability and the relative magnetic permeability of the ferrite core of the coil, *d* is the distance between the center of the main coil axis and the current wire, *I_STIM_* is the amplitude of the current square pulses, *t_RISE_* is the rise time of the square pulses, and $\delta_{k}$ is the angle between the surface normal of the kth coil loop and the magnetic field lines that concatenate with the loop at distance *d* (in principle, this angle changes for coil loops as they are arranged along a straight line while the field lines are circular).

If the length of the coil is small enough with respect to the distance from the current wire, $\cos\delta_{k}$ can be assumed as constant (cosδ). Hence, the EMF amplitude can be expressed as:

(2) $$\begin{array}{c} \left|\mathrm{EMF}\right|=\frac{\mu_{0}\mu_{r}}{2\pi }NS\cos\delta \;\frac{I_{STIM}}{t_{RISE}}\;\frac{1}{d}\; \end{array}$$

It can be observed that with a coil positioned at a fixed distance from the current wire on the measurement plane, the amplitude of the EMF can be adjusted both by increasing the amplitude of the current pulses and reducing the rise time. This feature allows obtaining reasonable EMF amplitudes even with low current pulse amplitudes, i.e., low pulse voltages across the wire, which is important to prevent undesired neuro-muscular stimulations when applied to an electrocatheter inside the human body. To this aim, the use of short pulses is also of help, as it avoids undesired stimulations even for higher voltage amplitudes, which can be purposely adopted to increase the amplitude of the measured EMF further. The materials and geometry of the coil can also be designed to optimize the amplitude of the measured EMF, e.g., by increasing the surface area and the number of the coil loops, which, however, would lead to an increase in the overall size of the coil.

Another important aspect to remember is that a coil has parasitic capacitance and resistance, thus behaving like an RLC circuit with an oscillatory impulse response. Therefore, the voltage signal measured on a coil due to the magnetic field generated by a wire with a square pulse current consists of two damped sine waves, one starting with a positive wave, the other with a negative wave, corresponding to the rising and falling edges of the square current pulses [24].

**Figure 3:**
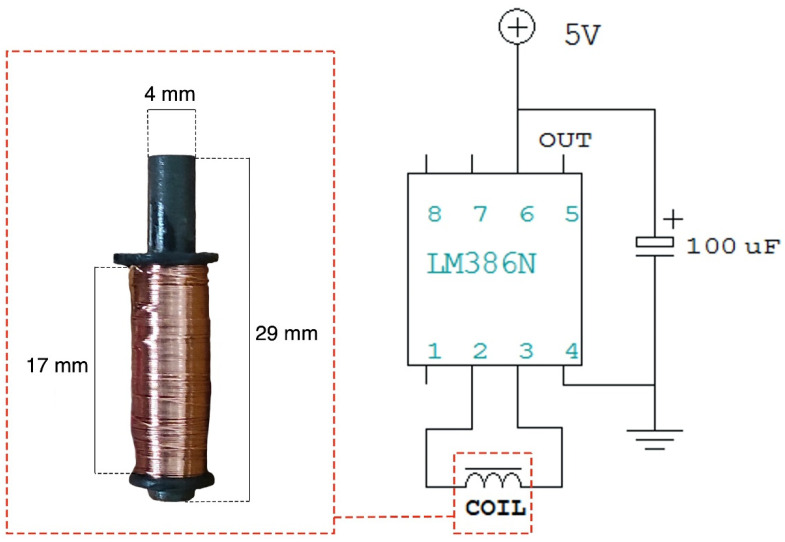
Schematic of the coil conditioning circuit with details on the actual coil used in the experiments and its geometrical parameters.

The EMF induced on the coil used in this study was amplified via the conditioning circuit depicted in Figure 3. The conditioning circuit consisted of an LM386 low-voltage power audio amplifier, which was set to provide a gain of 20 V/V. The coil voltage measurements were performed with a Fluke 123 Industrial Scopemeter oscilloscope (Fluke Corporation, 6920 Seaway Blvd, Everett, WA, USA).

### 2.3. Test on Copper Wire in Air

**Figure 4:**
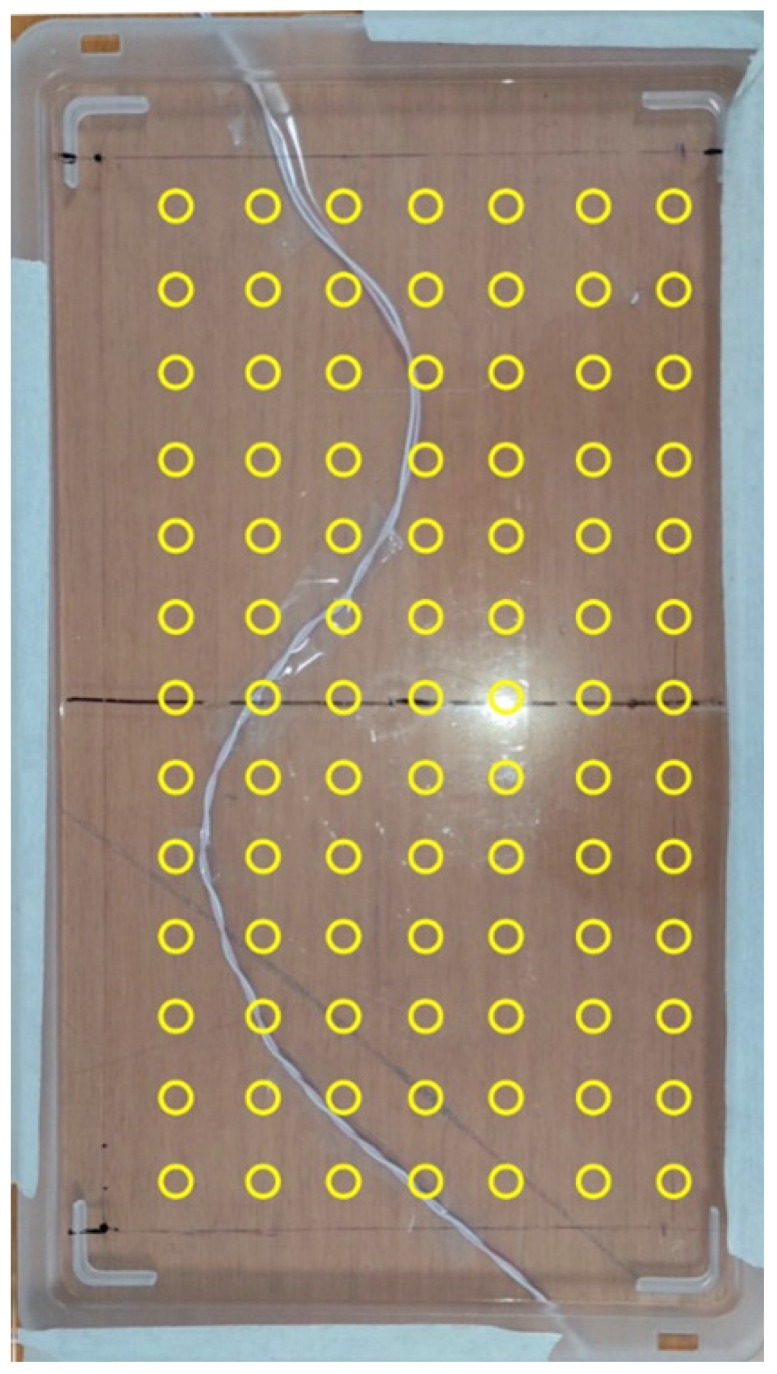
Measurement setup for the test on the copper wire. The yellow circles correspond to the measurement points.

A copper wire was laid on a wooden table. The wire was bent in such a way as to describe generic curves and was glued to the tabletop. A transparent plastic stand (33 × 19 cm) was placed one centimeter above the wire on a plane parallel to the table. A 7 × 13 grid of landmarks (7 on the *x*-axis, 13 on the *y*-axis) with a spacing of 2 cm in both directions was drawn on the plastic stand to allow accurate positioning of the coil. The measurement setup for this test is depicted in Figure 4. A function generator (HP 33120A, Hewlett-Packard Inc., 1501 Page Mill Road. Palo Alto, CA 94304, USA) was set to provide square pulses of 200 µs duration and 4V peak-to-peak amplitude. The voltage pulses were applied to a 500 Ω resistor (representative of the typical electrode-tissue resistance for pacemaker leads implanted in the human body) to mimic a realistic current flow through the copper wire. For each point, two measurements were acquired by aligning the main axis of the coil along the *x* and *y* axes of the plane.

This measurement setup involved magnetic field measurements in air. As the relative magnetic permeability of air is almost equal to the that of the water, the results were still representative of measurements made in water. However, in this experiment, the test current was obviously confined within the wire, while in the case of an electrocatheter in the human body, the current would also flow in the body tissues towards the return electrode. For this reason, a further test was performed by using an actual electrocatheter immersed in a saline solution.

### 2.4. Test on Electrocatheter in Saline Solution

A tank of 16 × 28 cm was filled with a 0.027 M saline solution to simulate the electrical characteristics of a human torso (mean resistivity of 375 Ω·cm), according to the standard ISO 14117:2019 [25], which describes a similar device used for electromagnetic compatibility measurements of pacemakers, defibrillators, and other active implantable devices.

A stainless-steel plate with a large surface area (10.5 × 5 cm) placed on one side of the tank containing the saline solution was used as the return electrode. For possible future real-world application on patients, such a wide electrode could be a common disposable return electrode used for high-frequency surgery equipment.

A real bipolar pacemaker lead (St. Jude Medical CAW257763 2088/58 |IS-1 B|, St. Jude Medical Luxembourg Holding S.a.r.l., Saint Paul, MN, USA), representing a generic lead inserted inside the patient, was used for the experiments. Nonetheless, only one electrode of the catheter was actually used to close the circuit between one terminal of the function generator and the return electrode (which was obviously attached to the other generator terminal). This arrangement is equivalent to using the electrocatheter as a unipolar lead. The electrocatheter was suspended one centimeter below the water surface. The function generator was set as described in Section 2.3.

**Figure 5:**
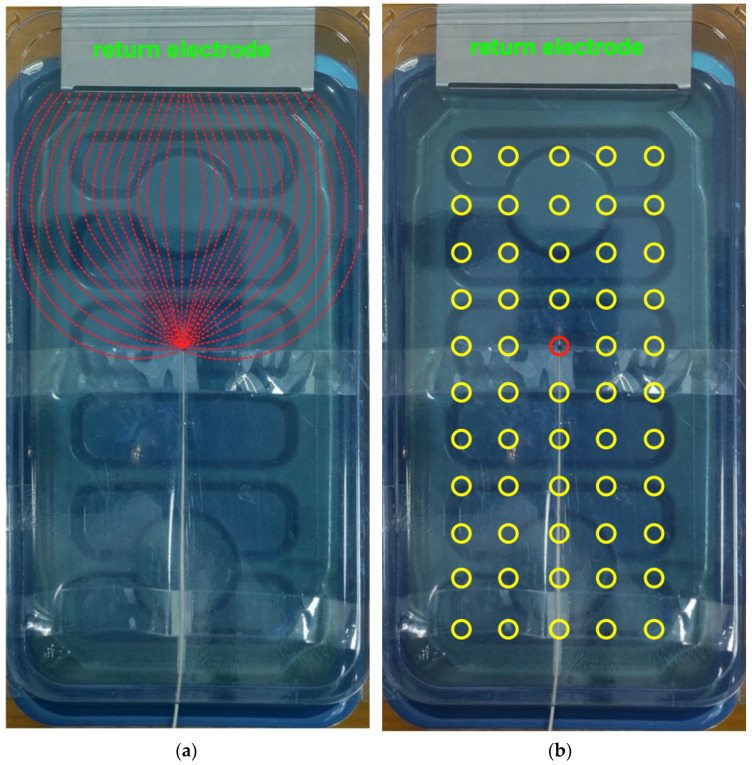
Measurement setup for the test on the electrocatheter in the saline solution. (**a**) The red dashed lines represent the main ionic current lines between the electrocatheter electrode and the return electrode; (**b**) the yellow circles correspond to the measurement points. The catheter electrode corresponded approximately to the red circle in position (3,5).

A transparent rigid plastic sheet was placed on the upper part of the tank containing the saline solution at 1 cm above the water surface, i.e., at 2 cm from the electrocatheter. A 5 × 11 grid of landmarks (5 on the *x*-axis, 11 on the *y*-axis) with a spacing of 2 cm in both directions was drawn on the plastic sheet to allow accurate positioning of the coil, as described in Section 2.3. The overall measurement setup for this test is depicted in Figure 5. In particular, Figure 5a illustrates the main ionic current lines between the electrocatheter electrode and the return electrode (red dashed lines), while Figure 5b shows the actual measurement points.

### 2.5. Data Processing

The measurements performed during the tests on the copper wire in air and the electrocatheter in saline solution produced couples of amplitude values matrices referred to as *V_x_* and *V_y_*, which had been obtained with the coil oriented along the *x* and *y* axes of the measurement plane, respectively. The amplitude values in *V_x_* and *V_y_* were proportional to the *x* and *y* components of the magnetic induction field vector. These matrices were processed to obtain an estimate of the current wire position (the copper wire and the electrocatheter). All the processing procedures were performed in MATLAB^®^ R2017b (The MathWorks, Inc., 1 Apple Hill Drive, Natick, MA, USA).

The *V_x_* and *V_y_* matrices were first interpolated with 2-D splines via the MATLAB^®^ function “*interp2*” using a uniform grid with a 0.05 cm spacing. Then, a matrix *V* containing amplitude values proportional to the modulus of the magnetic induction field vector was obtained from the interpolated matrices, referred to as $V_{x_{i}}$ and $V_{y_{i}}$, according to the following equation:

(3) $$\begin{array}{c} V\left(i,j\right)=\sqrt{V_{x_{i}}{\left(i,j\right)}^{2}+V_{y_{i}}{\left(i,j\right)}^{2}}\; \end{array}$$

The wire position was then estimated by first searching for the maximum value on each row of the modulus matrix *V*. A thresholding was then applied to the rows’ maxima, thus obtained, to exclude the contributions of magnetic induction fields much smaller than those generated by the lead (e.g., measurement noise). In particular, values lower than 70% of the absolute maximum value of the *V* matrix were excluded.

### 2.6. Theoretical Evaluation of Tissue Heating Effects

The energy associated with each individual pulse can be calculated from the amplitude and duration of the pulse and the resistance of the catheter-tissue interface. This resistance is about 500 Ω for a typical pacemaker lead. In our case, the pulse amplitude was set at 4 V and the duration at 200 µs.

We can therefore use the following formula:

(4) E=V·I·d

where *E* is the energy, *V* is the voltage, *I* is the current and *d* is the time duration of the pulse. Substituting the considered values in the formula, we obtain an energy of 6.4 µJ associated with each pulse. Let’s consider that in order to obtain a continuous video of the catheter, a frame rate of about 30 frames per second is needed; this means that 30 pulses per second are provided to the electrocatheter. From this, it follows that a power of about 192 µW is provided. The values obtained already guarantee a particularly low residual risk associated with the system. However, the rise in tissue temperature Δ*T* near the tip of the electrocatheter can be estimated by means of the bio-heat equation [26]:

(5) $$\Delta T=\frac{1}{\sigma \rho c}\;J^{2}\;t$$

where *σ* is the tissue electrical conductivity, ρ is the tissue mass density, *c* is the specific heat capacity, *J* is the current density, and *t* is the time interval considered.

The electrocatheter tip is in contact with blood, therefore, average blood parameters should be considered. The blood electrical conductivity is 0.61 S/m, blood density is similar to that of water, i.e., 1000 kg/m^−3^, and blood specific heat capacity is 3617 J/kg °C. The current density is maximum at the electrode-tissue interface; let’s consider an electrode surface of 1 mm^2^ and the current as the ratio of the applied voltage to electrode resistance (i.e., 4 V/500). Substituting these values in the bio-heat equation and considering a time interval *t* = 60 s, one obtains that the rise in temperature in one minute is about 10^−5^ °C. The computed temperature rise is certainly irrelevant in practical applications. Moreover, the bio-heat equation neglects the heat transport in the tissues. Indeed, the blood flowing through the vessels transports the heat away from the catheter, thus continuously cooling the catheter tip.

## 3. Results

### 3.1. Copper Wire in Air

**Figure 6:**
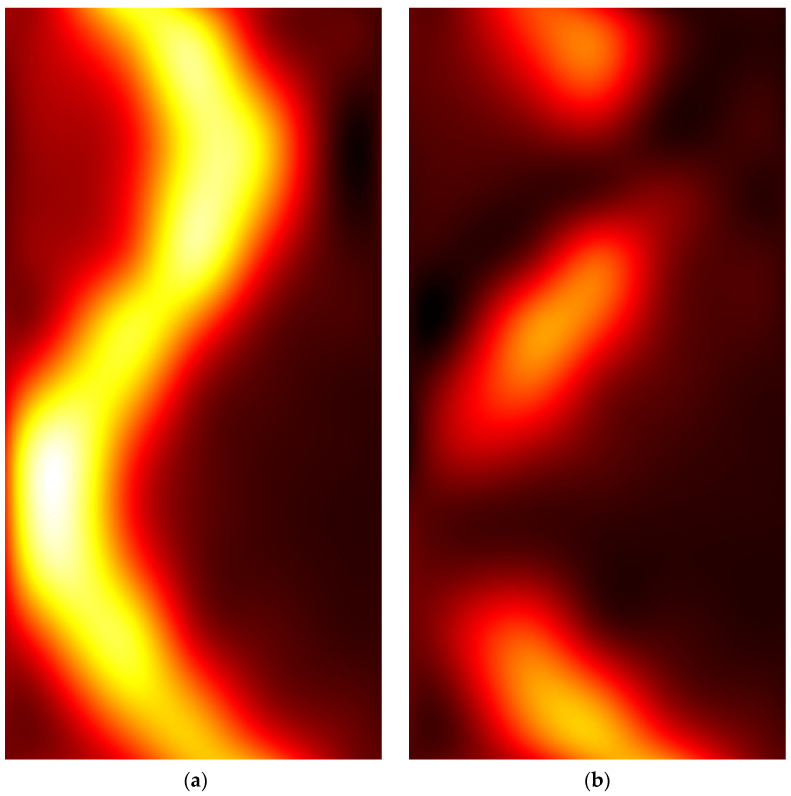
False-color images of the interpolated amplitude matrices were obtained from the copper wire with the coil aligned (**a**) along the *x*-axis ($V_{x_{i}}$) and (**b**) along the *y*-axis ($V_{y_{i}}$).

**Figure 7:**
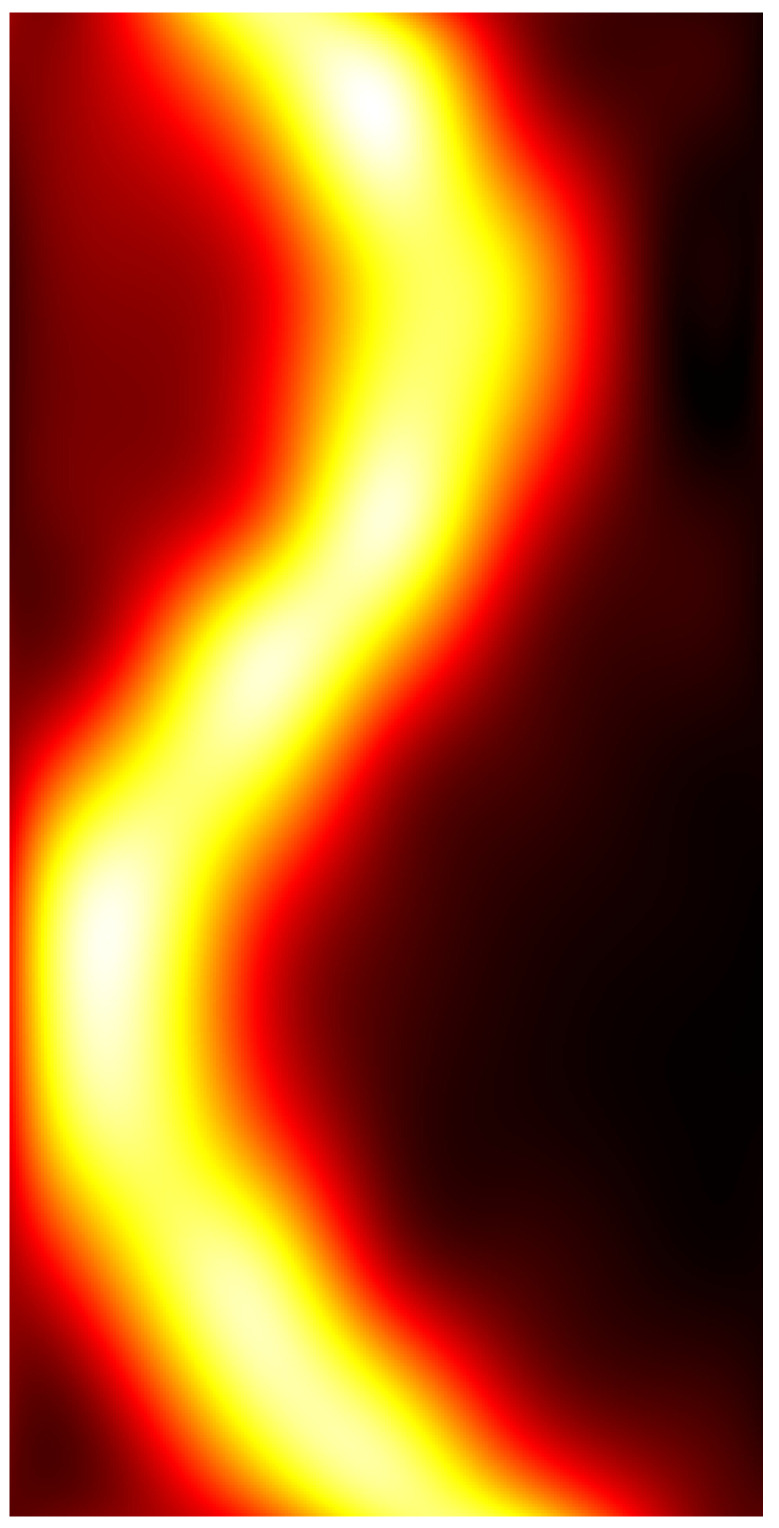
Pseudocolor image of the modulus matrix *V*, obtained from the $V_{x_{i}}$ and $V_{y_{i}}$ matrices depicted in Figure 6, by using the formula reported in Equation (3).

Figure 6 shows pseudocolor images representing the interpolated amplitude matrices $V_{x_{i}}$ and $V_{y_{i}}$ related to the copper wire in the air. It could be observed that a first guess of the wire shape can be obtained by visual inspection of $V_{x_{i}}$. However, by looking at the modulus matrix *V* depicted in Figure 7, which was obtained from $V_{x_{i}}$ and $V_{y_{i}}$ shown in Figure 6, a more accurate representation of the wire shape can be observed.

**Figure 8:**
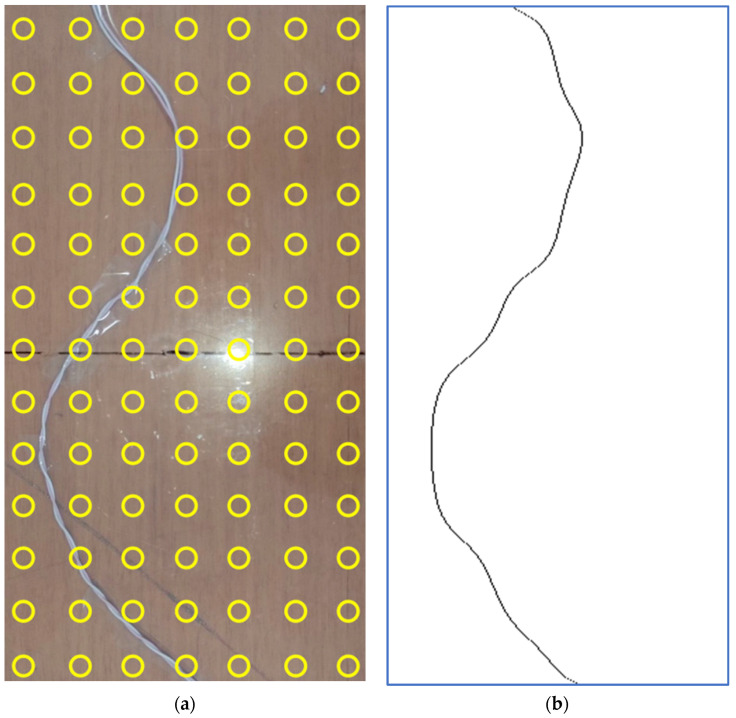
(**a**) Picture of the copper wire; (**b**) Estimation of copper wire position.

The estimate of the copper wire position, obtained by maxima search along the *V* matrix rows and the following thresholding, is depicted in Figure 8b, while Figure 8a shows a picture of the copper wire for visual comparison.

### 3.2. Electrocatheter in Saline Solution

**Figure 9:**
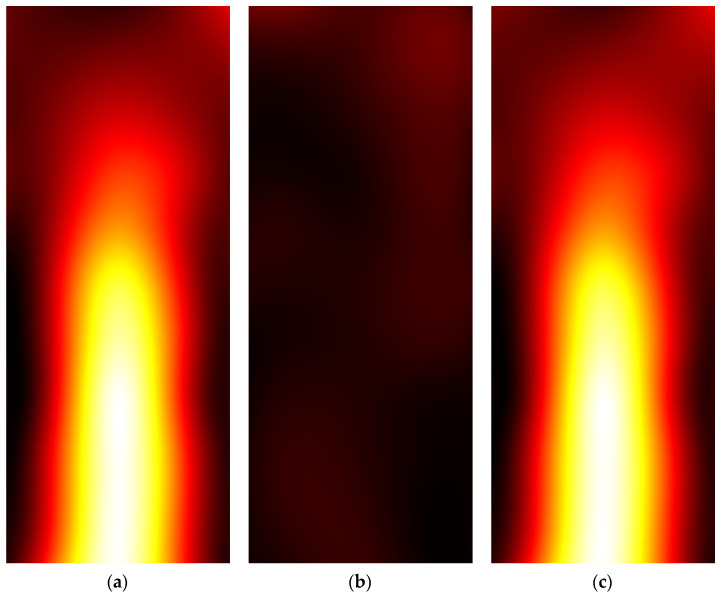
Pseudocolor images of (**a**,**b**) the interpolated amplitude matrices obtained from the electrocatheter with the coil aligned along the *x*-axis ($V_{x_{i}}$) and the *y*-axis ($V_{y_{i}}$); (**c**) the modulus matrix V obtained from the $V_{x_{i}}$ and $V_{y_{i}}$ matrices depicted in panels (**a**,**b**).

Figure 9 shows pseudocolor images representing the interpolated amplitude matrices $V_{x_{i}}$ and $V_{y_{i}}$ and the modulus matrix *V* related to the electrocatheter immersed in the saline solution. As expected, the *x* component represented the main contribution to the magnetic induction field generated by the electrocatheter.

**Figure 10:**
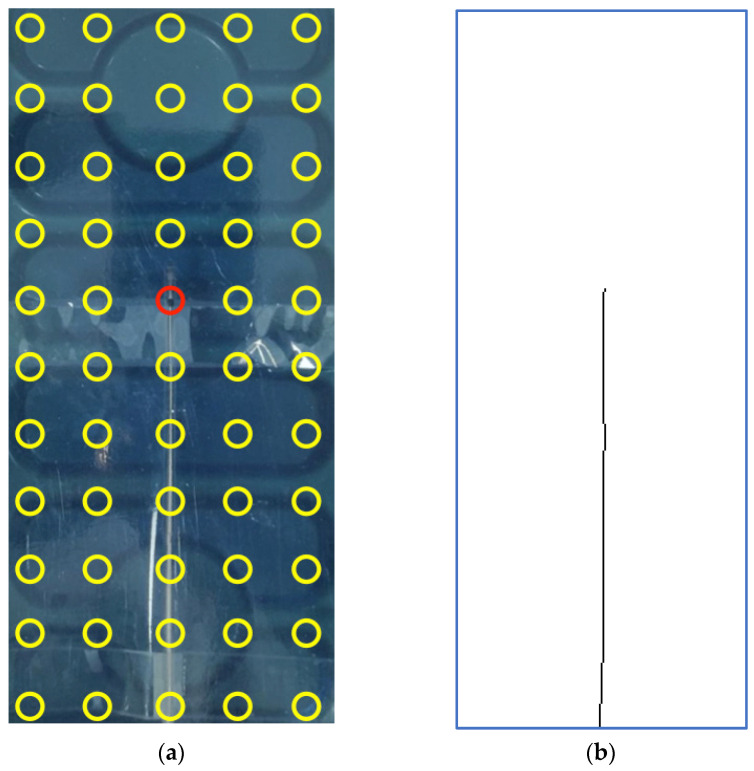
(**a**) Picture of the electrocatheter in the saline solution; (**b**) Estimation of the electrocatheter position.

Figure 10 shows a picture of the electrocatheter in panel (a) and the estimate of the electrocatheter position in panel (b). The estimated position of the catheter electrode was (4.15, 7.80) cm with reference to the top left corner of the measurement plane, while the position of the landmark where the catheter electrode was actually placed was (4, 8) cm. Therefore, a localization error of 2.5 mm was achieved.

## 4. Discussion

This study deals with the experimental verification of the hypothesis that it is possible to obtain accurate information on the positioning of a lead inside the human body by measuring the external magnetic field generated by a current flowing inside the lead itself.

The results obtained from the test on the copper wire confirmed the feasibility of accurately estimating the positioning of a bent wire. The test on the electrocatheter confirmed that the ionic current through the saline solution (flowing from the catheter electrode toward the return electrode) provided a much weaker contribution to the magnetic induction field, thus allowing to recognition of the catheter tip accurately.

These preliminary results confirm the starting hypothesis and invite us to continue with further and more detailed studies to propose this methodology as a possible alternative to the use of radiological equipment. The proposed methodology does not make any use of ionizing radiation, nor does it requires any instrumented catheter: this is a definite advantage over the current interventional practice. However, the proposed technique does not provide any anatomical detail as the radiographic technique partially allows. To overcome this limitation, it could be thought to superimpose the obtained image of the catheter into a camera image that frames the patient’s chest or some anatomical landmarks to show the surgeon concise and approximate information about the patient’s anatomy. Moreover, it is not to be excluded that the presented technique can be used in combination with fluoroscopy to reduce the radiation dose drastically.

For this preliminary study, we were limited to providing a projection of the catheter position on a plane. This was due to the desire to provide some equivalence with the fluoroscopic images used today. In the future, the estimation of catheter placement in three dimensions should be considered. To this aim, it may be considered to acquire measurements not only in the measurement plane but in multiple planes or on multiple curvilinear surfaces that would better accommodate the geometry of the patient’s body.

In addition, the magnetic field component on the *z*-axis, i.e., the one perpendicular to the measurement plane, was not taken into account in this preliminary study. Unlike the *x* and *y* components, the magnetic field measured on the *z*-axis theoretically should present nulls in correspondence with the projection of the leads on the measurement plane. Future experiments will include the measurement of the *z*-axis component.

The magnetic field strongly attenuates when moving away from the source. Therefore, the measurement plane should be placed not too far from the patient’s body. In the future, it would be useful to make experimental measurements to assess the maximum distance that would allow practical, accurate measurements of catheter placement. Other studies should also be carried out to optimize the choice of the measuring coil, its conditioning circuit, and the value of the background noise that practically limits an accurate measurement of the magnetic field.

Sudden variations of currents coming from the electrical system or from other electromedical equipment placed near the measuring plane generate signals on the coil similar in shape to those generated by the auxiliary current that is intentionally passed inside the lead. A potential solution to these problems could be synchronizing the signal acquisitions on the coils with auxiliary current pulses and predetermined pulse sequences that correspondingly generate a unique (or unlikely to be accidentally replicated) train of pulses.

Having at least bipolar coaxial catheters available, the return electrode could be avoided, and the auxiliary current can be channeled between the two electrodes of the catheter. By doing so, only the position of the catheter tract between the two electrodes should be recognized by monitoring the magnetic field, while the magnetic field around the catheter wire should be very weak because the currents flowing in the inner and outer conductors of the coaxial catheter would have the same amplitude but opposite direction, so the related magnetic fields should theoretically cancel out.

## Data Availability

Data are available upon reasonable request to E.A. and P.B.

## References

[1] Haghjoo M., Roka A. (2012). Techniques of Permanent Pacemaker Implantation. Current Issues and Recent Advances in Pacemaker Therapy.

[2] Al-Khatib S.M., Friedman P., Ellenbogen K.A. (2016). Defibrillators: Selecting the Right Device for the Right Patient. Circulation.

[3] Tsalafoutas I.A., Spanodimos S.G., Maniatis P.N., Fournarakis G.M., Koulentianos E.D., Tsigas D.L. (2005). Radiation doses to patients and cardiologists from permanent cardiac pacemaker implantation procedures. Pacing Clin. Electrophysiol..

[4] van Dijk J.D., Ottervanger J.P., Delnoy PP H., Lagerweij M., Knollema S., Slump C.H., Jager P.L. (2017). Impact of new X-ray technology on patient dose in pacemaker and implantable cardioverter defibrillator (ICD) implantations. J. Interv. Card Electrophysiol..

[5] Picano E., Piccaluga E., Padovani R., Antonio Traino C., Grazia Andreassi M., Guagliumi G. (2014). Risks Related To Fluoroscopy Radiation Associated With Electrophysiology Procedures. J. Atr. Fibrillation.

[6] Nair G.M., Nery P.B., Redpath C.J., Sadek M.M., Birnie D.H. (2016). Radiation safety and ergonomics in the electrophysiology laboratory: Update on recent advances. Curr. Opin. Cardiol..

[7] Cesarelli M., Bifulco P., Cerciello T., Romano M., Paura L. (2013). X-ray fluoroscopy noise modeling for filter design. Int. J. Comput. Assist. Radiol. Surg..

[8] Casella M., Dello Russo A., Russo E., Catto V., Pizzamiglio F., Zucchetti M., Majocchi B., Riva S., Vettor G., Dessanai M.A. (2018). X-Ray Exposure in Cardiac Electrophysiology: A Retrospective Analysis in 8150 Patients Over 7 Years of Activity in a Modern, Large-Volume Laboratory. J. Am. Heart Assoc..

[9] Eichenlaub M., Astheimer K., Minners J., Blum T., Restle C., Maring C., Schweitzer S., Thiel U., Neumann F.J., Arentz T. (2020). Evaluation of a new ultralow-dose radiation protocol for electrophysiological device implantation: A near-zero fluoroscopy approach for device implantation. Heart Rhythm.

[10] Andreozzi E., Fratini A., Esposito D., Cesarelli M., Bifulco P. (2021). Toward a priori noise characterization for real-time edge-aware denoising in fluoroscopic devices. Biomed. Eng. Online.

[11] Sarno A., Andreozzi E., De Caro D., Di Meo G., Strollo A.G.M., Cesarelli M., Bifulco P. (2019). Real-time algorithm for Poissonian noise reduction in low-dose fluoroscopy: Performance evaluation. Biomed. Eng. Online.

[12] Castellano G., De Caro D., Esposito D., Bifulco P., Napoli E., Petra N., Andreozzi E., Cesarelli M., Strollo A.G.M. (2019). An FPGA-Oriented Algorithm for Real-Time Filtering of Poisson Noise in Video Streams, with Application to X-ray Fluoroscopy. Circuits Syst. Signal Process..

[13] Gepstein L., Hayam G., Ben-Haim S.A. (1997). A novel method for nonfluoroscopic catheter-based electroanatomical mapping of the heart. In vitro and in vivo accuracy results. Circulation.

[14] Shpun S., Gepstein L., Hayam G., Ben-Haim S.A. (1997). Guidance of radiofrequency endocardial ablation with real-time three-dimensional magnetic navigation system. Circulation.

[15] Ruiz-Granell R., Ferrero A., Morell-Cabedo S., Martinez-Brotons A., Bertomeu V., Llacer A., García-Civera R. (2008). Implantation of single-lead atrioventricular permanent pacemakers guided by electroanatomic navigation without the use of fluoroscopy. Europace.

[16] Larsen T.R., Saini A., Moore J., Huizar J.F., Tan A.Y., Ellenbogen K.A., Kaszala K. (2019). Fluoroscopy reduction during device implantation by using three-dimensional navigation. A single-center experience. J. Cardiovasc. Electrophysiol..

[17] Guo P., Qiu J., Wang Y., Chen G., Proietti R., Fadhle A.S., Zhao C., Wen Wang D. (2018). Zero-fluoroscopy permanent pacemaker implantation using Ensite NavX system: Clinical viability or fanciful technique?. Pacing Clin. Electrophysiol..

[18] Qiu J., Wang Y., Chen G., Zhao C., Wang D.W. (2020). Progress in zero-fluoroscopy implantation of cardiac electronic device. Pacing Clin. Electrophysiol..

[19] Hua W., Liu X., Gu M., Niu H.X., Chen X., Tang M., Zhang S. (2021). Novel Wide-Band Dielectric Imaging System Guided Lead Deployment for His Bundle Pacing: A Feasibility Study. Front. Cardiovasc. Med..

[20] De Santis M., Cacciotti I. (2020). Wireless Implantable and Biodegradable Sensors for Postsurgery Monitoring: Current Status and Future Perspectives. Nanotechnology.

[21] Fernandes C., Taurino I. (2022). Biodegradable Molybdenum (Mo) and Tungsten (W) Devices: One Step Closer towards Fully-Transient Biomedical Implants. Sensors.

[22] Boutry C.M., Chandrahalim H., Streit P., Schinhammer M., Hänzi A.C., Hierold C. (2012). Towards Biodegradable Wireless Implants. Philos. Trans. A Math. Phys. Eng. Sci..

[23] Hosseini E.S., Dervin S., Ganguly P., Dahiya R. (2021). Biodegradable Materials for Sustainable Health Monitoring Devices. ACS Appl. Bio Mater..

[24] Andreozzi E., Gargiulo G.D., Fratini A., Esposito D., Bifulco P. (2018). A Contactless Sensor for Pacemaker Pulse Detection: Design Hints and Performance Assessment. Sensors.

[25] Active Implantable Medical Devices—Electromagnetic Compatibility—EMC Test Protocols for Implantable Cardiac Pacemakers, Implantable Cardioverter Defibrillators and Cardiac Resynchronization Devices.

[26] Pennes H. (1948). Analysis of tissue and arterial blood temperatures in the resting human forearm. J. Appl. Physiol..

